# INTEREST GROUP SESSION—ASSISTED LIVING: DEMENTIA IN ASSISTED LIVING: STATE VARIABILITY IN REGULATIONS, OVERSIGHT, AND RESIDENT OUTCOMES

**DOI:** 10.1093/geroni/igz038.2005

**Published:** 2019-11-08

**Authors:** Kali S Thomas, Lindsay Schwartz

**Affiliations:** 1 Providence VA Medical Center, Providence, Rhode Island, United States; 2 American Health Care Association/National Center for Assisted Living, Washington, District of Columbia, United States

## Abstract

Approximately one million individuals, an estimated 40% with a diagnosis of Alzheimer’s disease-related dementias (ADRD), reside in assisted living (AL); yet, little is known about their experience or the quality of care provided in AL. Unlike other forms of long-term care (LTC), the licensing, operating, and enforcement requirements for AL falls to the states, which vary dramatically in their regulatory approaches. The overall objective of this symposium is to examine states’ AL regulatory environments and understand if and how the health outcomes of AL residents with ADRD are impacted by states’ regulatory decisions. Presenters will highlight the state variability in the regulation, oversight, resident composition, and outcomes of AL residents with ADRD. The first presentation will describe states’ different regulatory requirements for staffing and admission/discharge criteria as it relates to residents with ADRD and how those have changed over the last decade. The second presentation will report results from a national survey of state agents regarding their oversight and enforcement activities in AL. The third presentation will characterize differences in the resident composition and healthcare utilization among residents with ADRD across states. The fourth presenter will report on the effect of residing in an AL licensed to provide specialized dementia care versus a standard-licensed AL on ADRD residents’ outcomes. The discussant will contextualize findings as they relate to the current state of the AL industry. Results will ultimately inform policy-makers, organizational leaders, and clinicians as they seek the most effective ways to ensure optimal outcomes vulnerable residents with ADRD.

